# Pathological Mechanisms and Novel Testing Methods in Thrombotic Thrombocytopenic Purpura

**DOI:** 10.3390/biomedicines12030621

**Published:** 2024-03-11

**Authors:** Hallie H. Dolin, Robert W. Maitta

**Affiliations:** Department of Pathology, University Hospitals Cleveland Medical Center, Case Western Reserve University, Cleveland, OH 44106, USA; hallie.dolin@uhhospitals.org

**Keywords:** thrombotic thrombocytopenic purpura, immunity, autoimmunity, platelets, coagulation

## Abstract

Thrombotic thrombocytopenic purpura (TTP) is an uncommon, but potentially disabling or even deadly, thrombotic microangiopathy with a well-studied mechanism of ADAMTS13 deficiency or dysfunction. While established treatments are largely effective, the standard ADAMTS13 testing required to definitively diagnose TTP may cause delays in diagnosis and treatment, highlighting the need for rapid and effective diagnostic methods. Additionally, the heterogeneous presentation and varied inciting events of TTP suggest more variation in its mechanism than previously thought, implying three potential pathways rather than the accepted two. The recent discovery of ADAMTS13 conformation as a potential contributor to TTP in addition to the proposal of using the absolute immature platelet count (A-IPC) as a biomarker, present novel areas for monitoring and treatment. A-IPC in particular may serve as a more rapid and accurate diagnostic test to distinguish TTP from non-TTP TMAs and to monitor treatment response and relapse. These considerations highlight the need to further study TTP in order to improve best practices and patient care.

## 1. Introduction

Thrombotic thrombocytopenic purpura (TTP), a severe and often deadly disease, has been extensively studied over the past several decades. The widely accepted mechanism of TTP involves the deficiency of congenital and/or antibodies to immune-mediated a disintegrin and metalloprotease with a thrombospondin type I motif, member 13 (ADAMTS13) [1,2]. The lack of ADAMTS13 leads to an inability to cleave ultralong von Willebrand factor (UL-vWF) multimers, which aggregate with platelets to create microthrombi [1,2,3]. This leads to thrombotic microangiopathy (TMA) and characteristic symptoms of TTP. 

While potentially catastrophic, TTP is difficult to diagnose, even as time is of the essence in its treatment. The “classic pentad” of hemolytic anemia, thrombocytopenia, fever, neurological symptoms, and kidney damage is uncommon and unhelpful since it is present in a single-digit percentage of patients [3,4]. The nervous system is the most common system affected, with neurological symptoms occurring in 40–80% of TTP patients; major sequelae, such as stroke, can be fatal [4]. Additional systems may be affected, including but not limited to the kidneys, skin, and gastrointestinal tract [4,5]. However, these symptoms overlap extensively with various TMAs in addition to any number of medication reactions, infections, autoimmune diseases, and/or neoplasias [3,5,6,7]. Complicating these differential diagnoses is the 90% mortality rate of untreated TTP requiring a high level of suspicion and testing which at times could be delayed [3]. 

These problems, and the possibility that the mechanism of TTP may be more heterogeneous than previously thought, continue to challenge practitioners and researchers alike even in an era of increasing communication and research capabilities. It is clear that in light of these difficulties, TTP must be further evaluated and classified in order to more effectively diagnose and treat it. Studies within the past decade have suggested laboratory measurements—most notably, immature platelet fraction (IPF) and absolute immature platelet count (A-IPC) tests may serve as complementary to ADAMTS13 testing, which for many institutions requires sending out testing to be performed at specialized reference laboratories, which is not ideal for a timely diagnosis [8,9,10,11,12]. Conversely, and with interesting implications for TTP pathophysiology, conformational changes in ADAMTS13 have been shown to contribute to the disease and may serve as a biomarker of disease progression or recurrence [13,14,15,16]. 

Further treatment is based on the subtype of TTP, which has been heretofore divided into immune-mediated (iTTP) and congenital (cTTP) forms, the former of which comprises 95–97% of TTP cases with cTTP representing the difference [17]. Approximately one-third of TTP cases are caused by cTTP in pregnant patients and children, although cTTP will occasionally present in adult patients as well; the mechanism mediating this has not yet been extensively studied [2,17]. In recent years, caplacizumab, a monoclonal antibody against the A1 domain of von Willebrand factor (vWF), has been developed and utilized for both iTTP and cTTP. However, this monoclonal antibody neither fixes the underlying ADAMTS13 deficiency nor eliminates the tendency towards antibody formation [2,17]. Furthermore, the possibility exists that with standard therapeutic plasma exchange (TPE) treatment, cTTP patients could develop antibodies to ADAMTS13 after exposure to plasma, thereby creating an iTTP-like state in addition to cTTP, thus complicating treatment [2]. This emphasizes the need to fully understand the underlying mechanisms mediating TTP in order to make treatment more precise. 

The importance of ADAMTS13-vWF interaction in TTP, whether in iTTP or cTTP, is highlighted by animal data. Multiple models have demonstrated that ADAMTS13 knockout or inhibition creates a TTP-like state when animals are injected with recombinant human vWF [18]. Given what is known of the disease, the proportions of its subtypes, and known interactions with vWF, further animal models will help to elucidate more accurately the pathophysiology of TTP subtypes. In short, newer data regarding TTP and its potential diagnosis and treatment can enhance knowledge of the disease, with the potential to streamline and improve diagnosis and treatments alike. 

## 2. Known Mechanisms, Laboratory Values, and Treatment 

The widely accepted mechanism of TTP begins with a disruption in normal regulation of the coagulation process due to decreases in ADAMTS13. A major function of ADAMTS13 is as a negative regulator of early coagulation, namely cleavage of UL-vWF multimers, thereby disallowing the formation of platelet-vWF thrombi in the small vessels thus maintaining homeostasis [1,2,3,4,5,7]. The congenital lack of, or autoantibodies against, ADAMTS13 depletes its levels in circulation and leads to the formation of platelet-multimer thrombi. This creates the characteristic phenomena of simultaneous decrease in platelet count, thrombi-induced blockage of small vessels, and hemolytic anemia due to mechanical effects on red blood cells [1,2,3,4,5,7,19]. Activation of the complement system, most notably the alternative complement pathway, as well as numerous other factors unfolding from the primary mechanism further contribute to the pathological process [20,21]. 

Platelet count < 30 × 10^9^/L is considered suspicious of TTP. Clinical scores have been developed to aid in the diagnosis of patients. One such score is the PLASMIC score, which takes into account platelet count, hemolysis variables, lack of active cancer within the past year and of solid organ or stem cell transplant, mean corpuscular volume (MCV) < 90 fL, international normalized ratio (INR) < 1.5, and creatinine < 2.0 mg/dL [5,22]. The French score, in contrast, measures three points: the same platelet count as the PLASMIC score (30 × 10^9^/L), creatinine < 2.26 mg/dL, and positive antinuclear antibody [23]. Of the two, the French score appears to be more sensitive and specific for TTP diagnosis, although both scores decrease in sensitivity and specificity with increased patient age, and positive predictive value is low in general TMA patient groups if proteinuria is not taken into account [24,25]. These shortcomings may be due in part to their use in groups of TMA patients with a high TTP prevalence, thereby lowering the accuracy of both scores [25]. 

Values < 20 × 10^9^/L are not uncommon platelet counts for TTP patients [3]. The possible reasons for these values are twofold: the known aggregation of platelets within thrombi that decreases platelet count, and importantly, the potentially insufficient production of functional platelets [8,9,10,11,12,19]. As such, platelet maturity has been proposed and studied as a potential biomarker for the disease and will be discussed later. 

**Figure 1:**
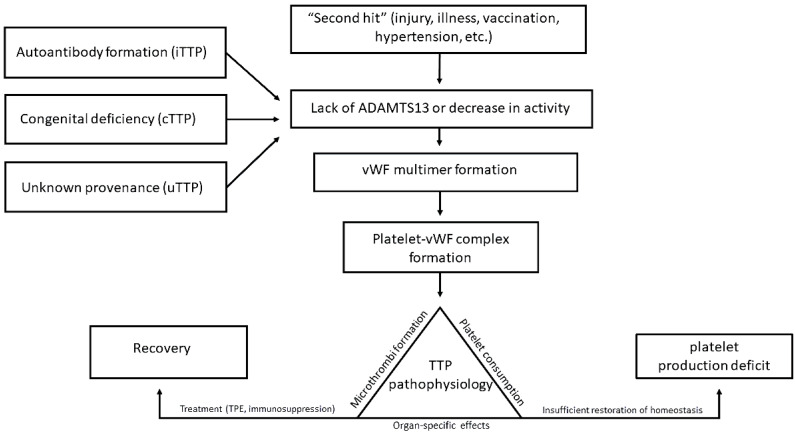
A simplified overview of known or suspected TTP pathologic processes. Included are immune, congenital, and unknown variants. A “second hit”, such as illness (infection), endothelial damage may predispose patients to disease. The dysfunction or lack of ADAMTS13 causes the uncontrolled formation of vWF multimers which leads to platelet–multimer aggregates in the microvasculature, creating the principal pathophysiologic mechanisms responsible for TTP. Treatment, such as therapeutic plasma exchange (TPE), corticosteroids, or rituximab +/− caplacizumab, leads to recovery if initiated promptly. Of note, these processes occur in the setting of immature platelet production deficit.

Endothelial cell damage and activity have been recently implicated as part of the pathophysiology of TTP, including but not limited to complement activation [19,20,21]. The “second hit” hypothesis posits that ADAMTS13 deficiency or a lack of activity is not sufficient to induce TMA, including TTP; rather, an additional stressor such as endothelial cell disruption, hypertension, or an unrelated disease state (Figure 1) are required for full onset of the disease [11,19,21]. These potential second hits can be varied and include but are not limited to pathogens, vaccinations, autoimmune disease, and possibly malignancy; certain HLA alleles also serve as a predisposing factor [21,26]. Notably, the COVID-19 vaccine in particular has been implicated in the development of TTP, in addition to the virus itself [27,28]. 

While varying causes of TTP contribute to its development, laboratory values are essential to its accurate diagnosis. Values reflecting the action of the coagulation cascade, most notably prothrombin time, activated partial thromboplastin time, and international normalized ratio, are within or slightly elevated above reference values, in contrast to disseminated intravascular coagulation (DIC) [3,4]. However, fibrinogen and lactate dehydrogenase do not differ significantly between TTP and DIC, and hemolytic uremic syndrome (HUS) presents with similar coagulation values; TTP and HUS may be distinguishable only by ADAMTS13 activity [3,29]. Misdiagnosis of TTP in a HUS patient can be problematic, as the standard TTP treatment (TPE) may not help HUS patients and at times worsen symptomatology [6,29]. 

TPE is the most effective treatment for TTP, and as such it is an essential part of the standard of care [1,4,17]. The procedure is effective for two reasons: it removes anti-ADAMTS13 antibodies from the affected patient’s circulation and replenishes ADAMTS13 stores with donor plasma allowing UL-vWF multimer cleavage [8]. In terms of treatment, given the overwhelming preponderance of iTTP, immunosuppression is routinely initiated with corticosteroids and/or the anti-CD20 monoclonal antibody rituximab [1,7,16,17]. 

Recent reports have highlighted an additional potential mechanism resulting in TTP that is unrelated to the congenital deficiency of or antibodies to ADAMTS13. Changes in ADAMTS13 to a “closed” conformation have been linked to a non-immune form of TTP, in a proposed phenomenon of TTP of unknown pathophysiology, or uTTP (Figure 1) [2,13,14,15,16]. In these cases, cTTP is not present, and antibodies to ADAMTS13 are not detected, leading to this third category [13,14,16]. While the open conformation is linked to iTTP, non-immune uTTP patients present with closed ADAMTS13 [14]. Furthermore, while autoimmune disease is often associated with iTTP, uTTP is associated more often with malignancy [14]. Nevertheless, further studies are necessary to determine the existence, inheritance, or full mechanism of uTTP as a cohesive entity. These discoveries imply new directions for biochemical study and TTP treatment. 

## 3. Potential New Pathologic Mechanisms in TTP 

### 3.1. Mechanisms and Prevalence of Conformational Change

Antibodies against ADAMTS13 may bind to different domains of the molecule. The spacer domain specifically is often a target for antibodies and is in fact the most common epitope against which anti-ADAMTS13 antibodies develop [2]. In normal circulation, ADAMTS13 circulates in a globular or “closed” conformation, kept so by interaction between three different domains [14]. However, upon stimulation with antibodies, the protein changes to an “open” conformation, fully exposing the spacer domain in addition to other regions as neo-epitopes to which antibodies may bind [2,17]. 

Interaction with vWF is known to induce the open conformation; although in a normal state, this occurs for only a short time [14]. Animal models also indicate that in iTTP, low levels of ADAMTS13 can be attributed to the clearance of immune complexes composed of antibodies and ADAMTS13 [18]. Whether this is associated with a certain length of conformational change has—as in humans—yet to be studied. 

Platelet count is higher in uTTP patients, averaging 28 × 10^9^/L versus 15 × 10^9^/L for iTTP [14]. Creatinine is significantly elevated in uTTP patients as well, averaging 183 µmol/L or 2.07 mg/dL in uTTP patients and 107 µmol/L or 1.21 mg/dL in iTTP patients; this implies potentially increased renal damage in uTTP. Pregnant patients with uTTP show higher blood pressure than patients with other forms [15]. Lastly, ADAMTS13 is in the closed conformation in 86.4% of uTTP cases but only in 23.3% of iTTP cases [13,14]. 

However, these results are not universal in uTTP. Pregnancy-induced TTP provides a potential area of study for the intersection of, and difference between, iTTP and uTTP. The presence of TTP variants and the known potential for their conflation creates areas of ambiguity under these circumstances [2,15,17]. Notably, pregnancy-induced uTTP shows a largely open conformation, in contrast to the closed conformation observed otherwise [14,15]. The relapse risk of pregnancy-induced uTTP also appears low, with only one relapse in the surveyed patients, as opposed to relapse in 23% of pregnant iTTP patients [15]. 

While the measurement of ADAMTS13 activity typically requires a send-out test, it may be possible to develop tests that assay the conformation of ADAMTS13 without having to wait for results from another institution [8,9,10,11,12,14]. Thus far, ELISA for ADAMTS13 has been used for this purpose [14]. However, given the known potential antibody binding sites on ADAMTS13 leading to iTTP, the development of assays specifically to detect the spacer domain and other areas important in maintaining conformation may also be desirable [2].

### 3.2. ADAMTS13 Interactions with Inflammatory States and Endothelial Damage 

Endothelial damage has previously been identified as a potential major player in TMAs as a whole, including TTP; this is evidenced by, for example, the ability of hypertension to induce TMA [11,19]. The increased potential for thrombosis when elements of Virchow’s triad (hypercoagulable state, endothelial damage, and blood stasis) occur in a patient is well known [30]. These factors increase the risk of TTP in, for example, COVID-19 infection, although there may be confounding factors if stasis due to immobilization rather than the virus is an equally causative agent in hospitalized patients [30]. Furthermore, while the exact mechanism of TTP in cancer has not been fully explored, it has been shown that cancer-induced TTP does not respond well to TPE, which may be consistent with uTTP largely lacking antibodies to be removed [31]. 

The potential of endothelial damage and its resultant activation of vWF, within or without a known prothrombotic state such as cancer or autoimmune disease, to play a role in TTP brings to mind the question of whether it contributes to TTP. Namely, are UL-vWF multimers produced at supraphysiologic levels that cannot be reasonably cleaved by normal amounts of ADAMTS13 in its normal conformation, thereby explaining its closed state in these cases, or does a yet unknown mechanism induce ADAMTS13 dysfunction regardless of endothelial activation? Approximately 25% of non-immune TTP patients do not have a quantitative ADAMTS13 deficiency [11]. This phenomenon lends credence to the hypothesis that *relative* rather than *absolute* ADAMTS13 deficiency in the setting of abnormally high UL-vWF multimer production may contribute to an identical pathology without an identical mechanism. In other words, ADAMTS13 may be simply insufficient rather than deficient in some cases, which could at least partially explain a normal closed configuration. This represents a viable area of future research. 

vWF has been proposed as a method by which cancer cells achieve metastasis, in effect being carried to distant sites via attachment to vWF multimers [32]. Cancer is known to be a risk factor for thrombosis via platelet–leukocyte aggregates as well, and whether these factors play into uTTP can be a potential area of study [33]. Additionally, as caplacizumab works specifically on vWF, in theory it may be useful as a therapeutic agent for uTTP, in cases where standard immunosuppressive therapy does not address the underlying pathophysiology [2,17]. 

## 4. Absolute Immature Platelet Count and Potential Platelet Production Suppression 

The immature platelet fraction (IPF), or the proportion of immature platelets within the total count that are newly released from the bone marrow, has been shown to help in triaging patients with iTTP. Notably, the absolute immature platelet count (A-IPC) derives from it [12]. To explore the usefulness of IPF/A-IPC as biomarkers for TTP diagnosis and relapse, it will be necessary to investigate the mechanism of platelet decrease and determine in which ways TPE drives immature platelet recovery. 

The measure of IPF/A-IPC is, in effect, a real-time gauge of thrombopoiesis. It allows for the identification of overproduction, compensation, or suppressed production in response to a thrombocytopenia-eliciting insult which can be promptly obtained with a complete blood count. Immature platelets can be distinguished from mature platelets by higher RNA levels, greater biochemical activity, and larger size than mature platelets [11]. Abnormally low values of these young platelets below the reference range are seen in iTTP, something that is paradoxical given the consumptive characteristic of new-onset iTTP [8,10,34]. In addition to iTTP, IPF/A-IPC may prove useful in immune thrombocytopenia (ITP) and heparin-induced thrombocytopenia, as these disorders stem from antibody-mediated mechanisms [35,36,37,38,39]. 

Low IPF indicates the inability of the bone marrow to compensate for platelet consumption, while high IPF indicates competent marrow and the subsequent production of relatively large quantities of immature platelets [10]. Of note, low A-IPC correlated better with an iTTP diagnosis and is potentially indicative of the severity of presentation, since sustained low A-IPC is seen in iTTP patients presenting with a high inhibitor to ADAMTS13 [35]. Particularly, A-IPC identified specifically and with great sensitivity those cases in which the PLASMIC score was low which suggested a low likelihood of iTTP and those cases in which a high score suggested iTTP when in reality patients did not have the disease [33]. In this regard, the low immature platelet count has shown to be more specific and sensitive by itself compared to the PLASMIC score, identifying all cases in which ADAMTS13 deficiency would be present [8,34]. This appears to provide additional possibilities to the pathophysiology of iTTP as it pertains to insufficient new immature platelet production, suggesting that iTTP is a non-compensated thrombocytopenia at presentation [8]. Additionally, these low A-IPC values appear to be decreased to a greater extent in new-onset iTTP compared to relapsing disease [8]. 

Monitoring increases in A-IPC soon after TPE initiation also characterizes iTTP from non-iTTP responses, especially when those other TMAs have laboratory results similar to TTP [8,12,34]. In iTTP, A-IPC rises two-to-three-fold higher after TPE initiation, whereas the same rise is not observed in other TMAs, suggesting that this measurement allows for the prediction of those patients that respond to therapy [8,12,34,35,40]. As mentioned earlier, TPE is not as helpful in HUS if used as a first-line treatment [29]; thus, obtaining and monitoring A-IPC until send-out ADAMTS13 results are available can be potentially important [8,12,34]. Since treatment decisions must be made promptly, at times before ADAMTS13 activity measurements are available, obtaining A-IPC at time of admission could guide TPE initiation and decision to discontinue it as applicable [8,9,10,11,12,29,34]. A-IPC may also help identify TMAs appearing at the same time as iTTP, as has been known to occur with processes such as ITP [5,40]. Namely, should a patient’s A-IPC not experience the expected fold A-IPC increase, having A-IPC and platelet count instability even if the patient initially responded to treatment, could be suggestive of a superimposed or simultaneous non-iTTP process [40]. 

It should be noted that A-IPC monitoring also seems to correlate with the course of recovery and presence of relapse in pediatric iTTP patients, and A-IPC returning to baseline after increasing during therapy is evidence that recovery has occurred [8,12,35,40,41]. Consequently, in critically ill pediatric iTTP patients who require timely biomarkers for disease progression or lack thereof, A-IPC, if monitored regularly, has the potential to be of use under such circumstances [41].

## 5. Importance of Low A-IPC and Possible Additional Pathologic Insult

Without question, the thrombocytopenia seen in iTTP is significant and indicative of a profound pathologic mechanism which drives platelet consumption. The removal of ADAMTS13 by antibodies to the enzyme appears to represent the best-known pathologic mediator leading to the observed thrombocytopenia that characterizes iTTP [42]. These antibodies, as mentioned earlier, bind ADAMTS13 and prevent its enzymatic function against vWF multimers. The presence of these autoreactive antibodies, and the ADAMTS13 depletion they cause, explain the benefit of medications that treat the resulting peripheral thrombocytopenia. Caplacizumab, as the seminal example, works to prevent the further binding of platelets to vWF [43]. The bone marrow works as a feedback loop in which the quantitative deficiency of a particular blood cell in the periphery triggers, as in the case of erythrocytes, marrow activation to produce the deficient element. Specifically, anemia triggers kidneys to increase erythropoietin production to subsequently drive higher reticulocyte output by the bone marrow to compensate for the erythrocyte deficiency [44]. A similar feedback response is seen when a patient suffers a hypoxic insult. The stimulus triggers greater erythropoietin synthesis to stimulate the bone marrow to produce more cells to relieve the hypoxia via increased cellular oxygen transportation [45]. 

In iTTP, platelet feedback responses appear to be minimized or absent, as shown by patients with new-onset disease. At presentation, the number of immature platelets, which are equivalent to reticulocytes in the equivalent scenario, are markedly reduced to levels below the normal reference range [8,34]. This is contradictory, since it would be expected for thrombocytopenia to stimulate a robust bone marrow response with an increase in immature platelet production. Importantly, the hypoproduction of immature platelets in iTTP is reversed with the initiation of TPE. Reversal occurs more quickly, even after a single procedure, in patients with a high inhibitor to ADAMTS13. Additionally, even when length of stay is taken into account, high inhibitor patients require a greater number of TPE procedures to overcome their disease, stabilize their platelet count, and achieve remission in both adult and pediatric settings [8,41]. 

**Figure 2:**
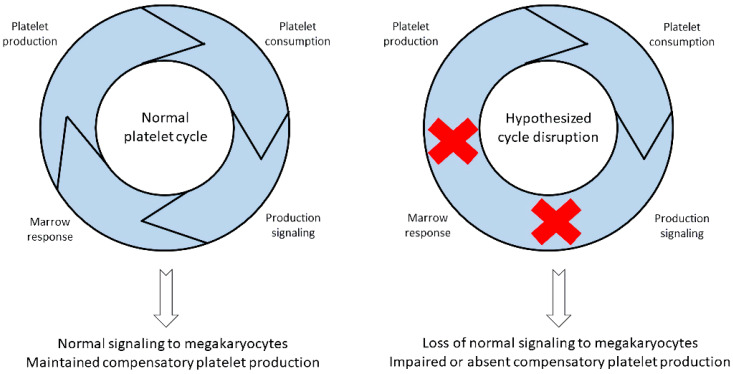
A schematic of the hypothesized relationship between changes in platelet production signaling, TTP, and dysfunction of compensatory immature platelet production. These production deficits suggest ADAMTS13-independent mechanisms. Left panel represents normal platelet homeostasis in which platelet production reciprocally increases during platelet consumption or count decreases. To the right is a possible unknown mechanism, or mechanisms, responsible for the inability of the bone marrow to compensate for platelet depletion during new-onset TTP. Red Xs are two potential areas that could explain the impaired immature platelet response by either improper signaling to the bone marrow to trigger platelet production or failure of bone marrow response to signaling.

Immature platelet hypoproduction indicates that the presence of potential defect(s) is at the level of the bone marrow [46]. Mechanistically, this occurs at the time of clinical presentation through a yet to be identified inhibitory process, which prevents the bone marrow from responding to the thrombocytopenia with higher immature platelet production (Figure 2). This is in addition to pathologic processes leading to ADAMTS13 decreases, either antibody-mediated or not. The observed inhibition or suppression of immature platelet production may take place at the time of antibody formation to ADAMTS13 or when the enzyme is being depleted below an activity level. Patients in remission who retain low ADAMTS13 levels, yet do not experience thrombocytopenia, suggest this mechanism [47]. Broad research into the pathophysiology driving the decrease in immature platelet production should be the focus of future investigations.

## 6. Other Contributors to TTP Pathogenesis and Outcome

Different aspects of the adaptive immune system, HLA alleles, and even blood groups may affect the tendency to contract TTP and/or respond to treatment. While evidence is conflicting, there appears to be differences in TTP pathogenesis, treatment response, and the risk of relapse depending upon whether the patient has blood group O or not. Type O blood appears to be protective against the development of TTP, possibly due to a tendency in those with this blood type to cleave vWF multimers more quickly or have lower levels of vWF at baseline [48,49]. However, while some data indicate that type O patients require fewer TPE sessions for full recovery, others have shown that type O patients require more TPE procedures and are increasingly at higher risk for relapse [48,49,50]. The common thread appears to be that there is a discrete difference between O and non-O blood groups without further differences in between, which could be in part due to the location of the ADAMTS13 locus adjacent to the ABO locus on chromosome 9 [51]. 

It has also been suggested that certain HLA alleles could increase or decrease the risk of TTP. The DRB-1*11 and DQB*03:01 alleles increase the propensity towards iTTP in Caucasian patients [17,21,26]. On the other hand, the DRB1*04 allele is considered protective in this group but is not protective in Japanese patients, while other alleles including DRB*08:03 are protective in the latter [17,21,26]. The mechanism is speculated to involve the easier binding of ADAMTS13-derived peptides, or HLA-binding peptides, to certain HLA alleles [21,26]. Nevertheless, the interaction of HLA and ADAMTS13 in relation to TTP requires additional research. 

The major contributors to conditions of excessive adaptive immune system activation, including but not limited to autoimmune diseases and TMAs, involve an interplay of cellular and humoral elements, prominently B and T cells of different subtypes. iTTP is an autoantibody-mediated phenomenon that functions as a Type II immune reaction, and certain aspects of lymphocyte populations—although heretofore not well studied—are characteristic. Recent research indicates that T-follicular helper (Tfh) cells have a yet to be determined role in TTP pathogenesis, and that furthermore, there could be defined differences in these cell populations between new-onset and relapsed iTTP [52]. In effect, new-onset TTP is characterized by the overexpression of and hyperfunction of the memory capabilities of Tfh cells. 

In contrast, during relapse, the primed immune system is less able to mount a response. This is especially evidenced by a decrease during relapse of double negative IgD^-^CD38^-^ memory B cells [52]. Similarly, activated plasmablasts are significantly higher in new-onset iTTP as compared to healthy controls but not in relapsed patients [52,53]. Thus, it has been hypothesized that increased plasmablasts increase the clearance rate of ADAMTS13 in new TTP patients due to first-time antibody production [52]. While the use of rituximab in relapsed patients may partially explain the lack of memory B cells, the higher A-IPC in relapsed versus new-onset iTTP does suggest that there is a mechanistic difference between iTTP types across immune responses [8,52,53]. 

How any or all of these elements contribute to uTTP is unknown, and they have not been studied in the context of immature platelet dynamics. However, it is important not to neglect any area of the immune system, blood cells, or cell-surface markers that may contribute to iTTP pathology, and all of the above are areas for future study. 

## 7. Conclusions

Thrombotic thrombocytopenic purpura is a complex, dynamic, and life-threatening disease with multiple known contributors and potential risk factors that remain to be fully understood. While ADAMTS13 deficiency and/or ADAMTS13 antibodies compose the widely accepted mechanism of TTP, such testing is often delayed compared to the need for treatment. Understanding the delicate divisions between potential subtypes, which may not all respond equally to the current standard of care, is vital for treatment optimization. 

Exploration of both the full mechanism(s) of iTTP and potential biomarkers that may distinguish iTTP from other TMAs as well as monitor treatment response is essential. Aspects of both the former and the latter have been reviewed in this paper, and more research into these subjects will provide new avenues for improvement of targeted therapy. If TTP diagnosis and monitoring can be streamlined and made more accurate, then the beneficial effects for patients would be immeasurable.
